# Dose-dependent improvement of myoclonic hyperkinesia due to Valproic acid in eight Huntington's Disease patients: a case series

**DOI:** 10.1186/1471-2377-6-11

**Published:** 2006-02-28

**Authors:** Carsten Saft, Thorsten Lauter, Peter H Kraus, Horst Przuntek, Juergen E Andrich

**Affiliations:** 1 Department of Neurology, Huntington-Center NRW, St. Josef Hospital, Bochum, Germany

## Abstract

**Background:**

Chorea in Huntington's Disease (HD) is usually treated with antidopaminergic neuroleptics like haloperidol, olanzapine and tiaprid or dopamine depleting drugs like tetrabenazine. Some patients with hyperkinesia, however, react to treatment with antidopaminergic drugs by developing extrapyramidal side effects. In earlier studies valproic acid showed no beneficial effect on involuntary choreatic movements. Myoclonus is rare in HD and is often overseen or misdiagnosed as chorea.

**Methods:**

In this report, we present eight patients whose main symptom is myoclonic hyperkinesia. All patients were treated with valproic acid and scored by using the Unified Huntington's Disease Rating Scale (UHDRS) motor score before and after treatment. In addition to this, two patients agreed to be videotaped.

**Results:**

In seven patients myoclonus and, therefore the UHDRS motor score improved in a dose dependent manner. In three of these patients antidopaminergic medication could be reduced.

**Conclusion:**

In the rare subgroup of HD patients suffering from myoclonic hyperkinesia, valproic acid is a possible alternative treatment.

## Background

Huntington's disease (HD) is an autosomal dominantly transmitted neurodegenerative disorder based on expansions of translated CAG repeats in the huntingtin gene beyond a threshold of 36 to >200 units. The characteristic motor feature of HD is chorea, but parkinsonism and involuntary movements such as dystonia and myoclonus can also be present. Choreatic movements are usually treated with antidopaminergic neuroleptics like haloperidol, fluphenazine, olanzapine or tiaprid and alternatively, dopamine-depleting drugs like tetrabenazine. Dopaminergic drugs or even low-dose levodopa can be administered in patients with the juvenile akinetic rigid variant of HD.

Myoclonus is a rare feature of HD. It does not sufficiently respond to antidopaminergic medication. A few reports of myoclonus in HD have mainly concerned cases of juvenile onset [1-3].

Earlier studies using valproic acid in HD did not show a beneficial effect on involuntary movements particularly with regard to choreatic hyperkinesias ([4] 2 patients; [5,6] 5 patients; [7] 14 patients; [8] 8 patients; [9] 3 patients; [10] 1 patient).

On the other hand, a few case reports describing myoclonus in HD report an improvement in movement disturbances after administration of valproic acid or a medication other than antidopaminergics. Carella et al. described a patient with adult-onset HD suffering from prominent action myoclonus. In this case, treatment with valproic acid greatly reduced myoclonus, suggesting that the gamma-aminobutyric acid (GABA) system might be involved in the pathophysiology of myoclonus in HD [11]. Another case report refers to two brothers with clinically diagnosed adult HD. Years after their first symptoms appeared, they developed disabling myoclonus, which could be brought under control by administration of valproic acid [12]. Thompson et al. describe three patients with HD who developed symptoms before the age of 30 with myoclonus as the predominant feature. The myoclonus improved under piracetam therapy in one patient and a combination of valproic acid and clonazepam in the others [13]. Kereshi et al. described a reduction of symptoms in a 26 year old woman suffering from HD with myoclonic hyperkinesias after a combined treatment with haloperidol and valproic acid [14]. Two further case reports describe an improvement of severe intention myoclonus by use of clonazepam in adult HD [15,1]. Funakawa et al. report on a cortical reflex myoclonus in adult onset HD. Oral administration of clonazepam was temporarily effective for myoclonus [16].

Furthermore, valproic acid has also been reported to be effective as a mood stabilizer in HD [17].

We herein describe eight adult HD patients suffering from severe action myoclonus or myoclonic hyperkinesia leading to physical disability. All patients were treated with valproic acid. To our knowledge, this is the largest group of patients exhibiting this rare feature to be reported thus far.

## Methods

About 90% of 600 HD patients investigated in our center during the last 10 years showed symptoms of chorea, about 60% suffered from choreatic movements as the main somatic symptom. Eight patients suffered from myoclonus as the main clinical symptom and were treated with valproic acid. Patients were scored by Unified Huntington's Disease Rating Scale (UHDRS) motor score before and after treatment with valproic acid [18,19]. Informed consent was obtained from each patient or the legal guardian. Two patients agreed to be videotaped before and after treatment with valproic acid. One patient was capable of performing a handwriting test before and after treatment with valproic acid. Peg insertion was performed during treatment in order to evaluate executive dysfunction and motor impairment, if possible [20,21].

All patients had been genetically tested and were symptomatic for HD with choreatic hyperkinesia. Additionally, all patients showed signs of akinesia and rigidity, most of them also suffered from dysphagia. Myoclonus, however, was the predominant clinical symptom. All patients demonstrated a worsening of myoclonus during action, some did also present severe myoclonic hyperkinesias at rest (case 1, 3, 6 and 7). All patients presented a multifocal positive myoclonus. In one patient (case 3) a stimulus sensitive myoclonus was observed, but movement also occurred while active. No additional electrophysiological studies were performed. One patient (case 3) had been treated with valproic acid due to seizures before first investigation (1050 mg/per day, serum level: 31 μg/ml) but the dose had to be increased during treatment. The same patient was readmitted 4 years later because of worsening of the symptoms and was treated again by increasing the valproic acid dosage (initial dose 1800 mg, serum level: 37 μg/ml). Characteristics of all patients are described in table 1.

**Table 1:** Clinical characteristics of the patients

Patient	Sex	CAG	AO Motoric	AO Psychic	Duration	TFC	IS %	Rigidity	Swallowing problems
Case 1	M	22/46	28	22	11	2	20	++	+++
Case 2	F	20/54	37	ND	6	5	70	+	-
Case 3*	M	17/48	23	23	8	3	40	++	++
Case 3*	M	17/48	23	23	12	2	30	++	+++
Case 4	M	19/48	33	ND	11	4	40	+++	++
Case 5	M	17/43	50	45	6	3	40	++	++
Case 6	F	25/52	31	ND	10	3	40	++	++
Case 7	F	17/50	28	26	7	6	70	+	-
Case 8	M	17/50	30	30	11	3	30	+++	+++

Clinical characteristics of the patients. AO = age of onset, TFC = Total functional capacity (UHDRS), IS = Independence score (UHDRS), CAG = CAG-ranges (low/high); Intensity: - = none, + = mild, ++ = moderate, +++ = severe. ND = no data. One patient (*) was treated twice with an increased dose of valproic acid within a 4 year period, see text.

## Results

In seven patients myoclonic hyperkinesias and, therefore, UHDRS scores improved in a dose-dependent manner (see table 2). Initial mean UHDRS motor score was 73.1 (± 11.9), after treatment mean UHDRS motor score was 60.2 (± 12.8) for all patients (p = 0.042; t-test; data showed a normal distribution according to the Kolmogorow-Smirnow test) due to an improvement in overall motor function. In three of these patients antidopaminergic medication could be markedly reduced (case 3, 6 and 7), in the remaining patients antidopaminergic treatment was basically unchanged (table 2). Especially in case 1 and 3, swallowing improved. One patient with a daily dose of only 300 mg valproate (case 4) did not improve. Changes in co-medication during treatment are presented in table 2.

**Table 2:** Clinical data and co-medication before and after treatment

Patient	Initial UHDRS motor score	Second UHDRS motor score	Valproic acid (mg/day)	Valproic acid serum level (μg/ml)	Mood stabiliza-tion	Improve-ment of mobility and manual dexterity	Changes in co-medication in mg
Case 1	80	72	900	30	+++	+	T100 CBZ900 MEL37.5 TE⇔, LO⇔ CL25
Case 2	65	33	1200	87	-	+++	R⇔
Case 3*	79	61	1800	59	+	+	T100 CL44 CLO0.5 LEV1000 TE⇔ CBZ400
Case 3*	84	74	2700	43	++	++	T300 TE25 CLO0.5 CL⇔ CBZ400
Case 4	56	56	300	(ND)	-	-	T⇔, TE⇔, LO⇔, OX⇔, HAL⇔
Case 5	72	66	900	73	++	+	T⇔, HAL⇔
Case 6	74	60	1050	43	+++	++	T150 TE25 S200 Q⇔
Case 7	57	50	1950	84	+	+++	T300 LO1.5
Case 8	91	70	1350	56	++	++	T50 LO⇔, Q⇔, MEL⇔

Clinical data and co-medication before (initial UHDRS motor score) and after (second UHDRS motor score) treatment with valproic acid. ND = no data; Intensity: - = no, + = mild, ++ = moderate, +++ = very good. Medication: T = Tiaprid, TE = Tetrabenazine, CBZ = Carbamazepine, CL = Clozapine, CLO = Clonazepam, MEL = Melperone, LO = Lorazepam, R = Rilutek, LEV = Levetiracetam, OX = Oxazepam, HAL = Haloperidol, S = Sulpride, Q = Quetiapin. Signs: Increase = , decrease =  no change = ⇔. One patient (*) was treated twice with an increased dose of valproic acid within a 4 year period, see text.

In five cases a remarkable mood-stabilizing effect of valproic acid could be observed. In case 2 myoclonic hyperkinesia improved very much due to treatment with valproic acid. Especially mobility and manual dexterity were improved. This is also illustrated by handwriting tests (see picture 1 without, picture 2 with 900 mg, picture 3 with 1200 mg valproic acid). Initially and after treatment with 600 mg valproic acid, the above-mentioned patient was not able to perform peg insertion. After treatment with 1200 mg valproic acid peg insertion was possible, however strongly impaired (319 s right hand, 481 s left hand). Videotapes were performed before and after treatment with valproic acid (video 1 [see Additional file 1] without and video 2 [see Additional file 2] with 900 mg valproic acid for case 1, video 3 [see Additional file 3] without and video 4 [see Additional file 4] with 1950 mg valproic acid for case 7). No relevant side effects were observed.

## Discussion

Due to specific degeneration of striatal neurons even HD-patients with hyperkinesia may develop extrapyramidal side effects during antidopaminergic therapy. Moreover, not only hyperkinetic involuntary movements but also bradykinesia has been increasingly recognized as one of the key symptoms also in early stages of the disease in non-juvenile HD-patients [22-24]. Therefore, antidopaminergic medication often leads to a worsening of swallowing problems and gait disturbances.

In a subgroup of HD patients suffering from myoclonic hyperkinesia, valproic acid may be a possible alternative treatment. Valproic acid seems to be more efficient than antidopaminergics in these patients. It has no side effects on the extrapyramidal system as opposed to neuroleptics. In some of our cases, reduction of antidopaminergic medication caused an improvement of bradykinesia and swallowing.

The effects of valproic acid seem to be dose-dependent as demonstrated in cases 2 and 3. In case 2, handwriting tests show a remarkable improvement after an increase of dosage (see pictures 1–3). Because of worsening of symptoms, one patient (listed as case 3) was treated twice within a 4 year period. An improvement of symptoms could be reached each time after increasing the dosage of valproic acid; this might be a further indication of dose-dependent effects. Only one patient (case 4) receiving a daily dose of only 300 mg valproic acid did not improve. This might be due to insufficient dosage. For all other patients the reduction of disabling myoclonus led to a significant improvement in daily life activity.

Almost all patients developed initial HD symptoms early in life (AO 23 years up to 33 years) but none of them was a juvenile HD patient with characteristic akinesia and rigidity. Myoclonus appeared 6 to 12 years after AO of first symptoms of the disease in all patients. Almost all patients had an expanded CAG range between 45 and 50. It appears that a subgroup of patients with predominant myoclonus exists between those with typical choreatic movements and those with the juvenile akinetic-rigid variant.

As Carella et al. postulated, the gamma-aminobutyric acid (GABA) system might be involved in the pathophysiology of myoclonus in HD [11]. Earlier animal models with stereotaxic injection of kainic acid into rat striatum produced neuronal degeneration and neurochemical alterations resembling HD [25]. Since then, it was assumed that correcting the deficiency in gamma-aminobutyric acid (GABA), may be of therapeutic value.

The postulated mechanisms for myoclonus in HD have differed in literature [26]. Neurophysiological investigations in most earlier studies, documented generalised and multifocal action myoclonus of cortical origin. In some cases, myoclonus was strikingly stimulus sensitive [13]. Myoclonus, as recorded by surface electromyography consisted of 40–60 ms-synchronous semirhythmic bursts. The cortical component of somatosensory evoked potential was enlarged in some cases, representing a cortical myoclonus [1,26]. Since additional electrophysiological studies in our cases were not performed we can only speculate about a presumed cortical origin of myoclonus following literature and our clinical impression.

Furthermore, there are some reports suggesting a neuroprotective role of valproic acid, acting as a histone deacetylase-Inhibitor [27-36]. Thus, valproic acid seems to be a promising candidate for a new approach to treating Huntington's Disease.

## Conclusion

In the rare subgroup of HD patients suffering from myoclonic hyperkinesia, valproic acid is a possible alternative treatment.

## Competing interests

The author(s) declare that they have no competing interests.

## Authors' contributions

CS has made substantial contributions to conception and design, acquisition of data and drafting the manuscript; TL made substantial contributions to acquisition of data; PK to analysis and interpretation of data; HP revising it critically for important intellectual content; JA has been involved in acquisition of data, drafting the manuscript and has given final approval of the version to be published.

**Figure 1:**
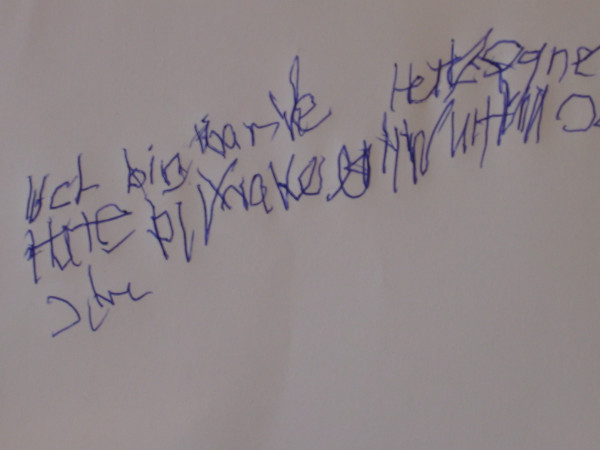
Case 2 handwriting test without vaproic acid treatment.

**Figure 2:**
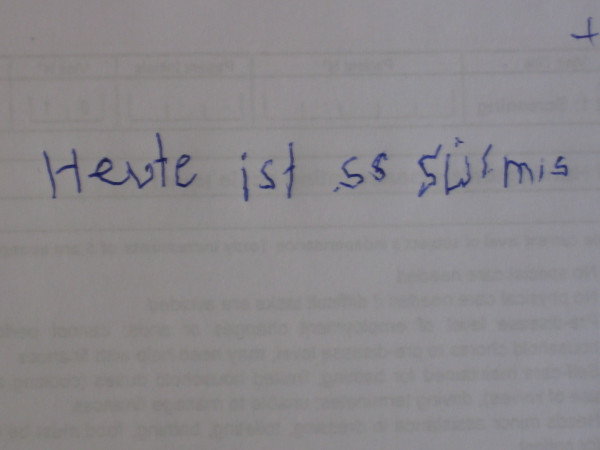
Case 2 handwriting test with 900 mg vaproic acid treatment.

**Figure 3:**
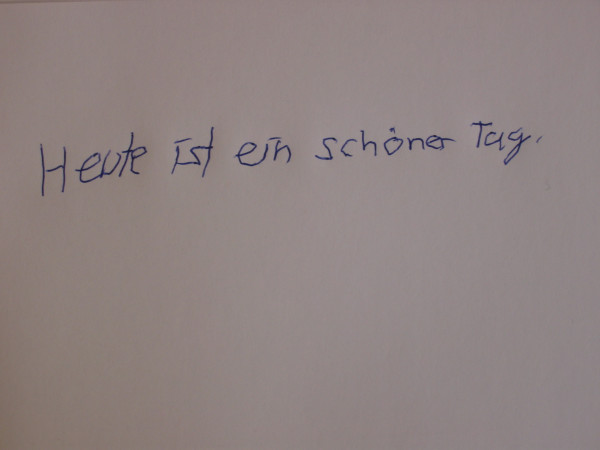
Case 2 handwriting test with 1200 mg vaproic acid treatment.

## Pre-publication history

The pre-publication history for this paper can be accessed here:



## Supplementary Material

Additional File 1 Case 1 without valproic acid treatment. Click here for file

Additional File 2 Case 1 with 900 mg valproic acid treatment Click here for file

Additional File 3 Case 7 without valproic acid treatment. Click here for file

Additional File 4 Case 7 with 1950 mg valproic acid treatment. Click here for file
